# Elaboration of Neuropsychological Evaluation of Children: Structural Analysis of Test Results

**DOI:** 10.11621/pir.2021.0402

**Published:** 2021-09-28

**Authors:** Aleksei A. Korneev, Ekaterina Yu. Matveeva, Tatiana V. Akhutina

**Affiliations:** a Lomonosov Moscow State University, Moscow, Russia

**Keywords:** Neuropsychological assessment, development ofhigher mental functions, primary school students, cognitive functions, face-to-face testing, computer testing

## Abstract

**Background:**

Modern neuropsychology is discussing the possibility of combining qualitative and quantitative approaches in the evaluation of cognitive functions. In Russia a battery of tests called “Methods of neuropsychological assessment for children 6–9 years old” (Akhutina et al., 2016) has been proposed; it is based on the Lurian approach to diagnosis and combines qualitative and quantitative approaches to testing. The present paper describes the development of this combined qualitative and quantitative assessment of various groups of cognitive functions in preschool and primary school children. Structural modeling enables us to analyze a possible combination of integral indices of functions that includes the results of both a face-to-face neuropsychological assessment and computerized testing.

**Objective:**

To develop a combined qualitative and quantitative neuropsychological assessment of children, in order to 1) check the structural reliability of integral indicators of various cognitive functions; and 2) confirm the correctness of combining the results of face-to-face and computerized tests.

**Design:**

A sample of 299 children between the ages of 6 and 9 years old (111 preschoolers, 82 first graders, and 106 second graders) underwent a Lurian face-to-face neuropsychological examination adapted for 6-to-9 year-old children, and five tests from the Computerized Neuropsychological Assessment for 6–9 Year-old Children. The five were the “Dots” test, the Schulte Tables, the Cancellation test, the Corsi Tapping Block test, and the Understanding of Similar Sounding Words test. In each of the tests (face-to-face and computerized), key parameters were identified to evaluate various cognitive functions.

**Results:**

A confirmatory factor analysis verified the composition of the neuropsychological indices that were based on the results of the face-to-face neuropsychological assessment. At the same time, when the computer test data were added to the model, the fit indices of the model considerably improved.

**Conclusion:**

The confirmatory factor analysis confirmed the validity of the identification of eight neuropsychological indices that indicate the component processes underlying complex cognitive functions in children: 1) programming and control of voluntary actions (executive functions); 2) serial organization of movements and speech; 3) the processing of kinesthetic information; 4) the processing of auditory information; 5) the processing of visual information; 6) the processing of visual-spatial information; 7) hyperactivity/impulsivity; and 8) fatigue/slowness.

## Introduction

Neuropsychological methods that examine and assess the state of cognitive functions in children are under development all over the world. Earlier stages of the development of such methods were characterized by two different approaches: a psychometric quantitative one, and a qualitative one based on the theory of functional systems. The first approach is based on expert-independent objective testing and quantification of results. The second is devoted to a detailed analysis of the quality of the subjects’ test performance. However, the present stage of child neuropsychology research is characterized by the convergence of these approaches for determining a diagnosis (Akhutina, Ignat’eva, Maksimenko, Polonskaya, Pylaeva, & Yablokova, 1996; Akhutina, 2016; Baron, 2004; Golden, 1987; Korkman, 1998; Korkman, Kirk, & Kemp, 2007; Reitan, 1959; Reitan & Wolfson, 1985; Tramontana & Hooper, 1988; Weiler, Willis, & Kennedy, 2019).

How were the qualitative and quantitative approaches combined in the research by T.V. Akhutina and her colleagues? If, as in making a qualitative diagnosis of adults, one can proceed from a general idea of the normative performance of tasks and take it as 0, then when working with children, it is necessary to quantitatively assess the qualitative characteristics of task performance by typically developing children of different ages. To capture the qualitative specificity of the test performance and be able to assess it quantitatively, it has been necessary to standardize the presentation and analysis of the tests, and to develop parameters for evaluating not only the productivity of test execution but also specific errors which reflect both primary and secondary difficulties in completing the tasks. The division of errors into primary and secondary types is a distinctive feature of neuropsychological analysis in the Vygotsky-Luria approach. Combining the productivity parameter for a certain test with the primary errors during its performance, and then summing them up with the parameters of other tests aimed at detecting the same primary defect, has led to the development of integral estimates, or indices (Korneev & Akhutina, 2016).

The method of adding the parameters of several tests has also been proposed within the quantitative approach; it is known as latent process analysis (Miyake, Emerson, & Friedman, 2000). To solve the problem of analyzing the component comprising executive functions, Miyake and his colleagues concentrated on “the task impurity problem.” Due to the complexity of any human activity, there are no tasks that are unambiguously associated with only one cognitive function without involving others. Latent process analysis makes it possible to reduce the impurity of tests. In discussing executive functions, the authors write: “A latent variable is essentially a hypothetical construct created by statistically ‘extracting’ what is common among the multiple tasks chosen to tap that construct. In the case of our study, we created three latent variables that corresponded to the Shifting, Updating, and Inhibition factors, respectively. It is important to point out that these latent variables were ‘purer’ measures of the target executive functions because each latent variable contained only what was shared among all three tasks and not what was specific to each task.” (Miyake et al., 2000, p. 178)

A similar method of adding unidirectional parameters to aggregate a set of results which would give a “purer” objective estimation has also been developed in child neuropsychology based on the Vygotsky-Luria theory. It is applied to assess not only executive functions but information-processing functions and functions of activation. This approach has evolved from the principles and methods of diagnosing local brain lesions in adults described by Vygotsky and Luria (Luria, 1965, 1976, 1980). It has turned out to be efficient even in solving such difficult issues in child neuropsychology as the analysis of cognitive functions for the selection of effective methods to overcome learning difficulties (Akhutina & Pylaeva, 2012).

In the context of the assessment of typical and deviant development, a further elaboration of optimal methods for evaluating cognitive functions in terms of efficiency, conciseness, and ecological validity turns out to be an important issue. In particular, we should examine a set of quantitative parameters of the quality of test performance, which we can use when calculating integral estimates or indicators of various cognitive functions. The need for such verification grows out of the problem of the “insufficient purity” of any test or task. To check the correctness of distributing the parameters into indices, we use the method of confirmatory factor analysis.

Within our work, a comprehensive neuropsychological examination for 6-to-9 year-old children was employed to diagnose the children’s state of higher mental functions (Akhutina, 2016). This battery consisted of 20 tests; their performance was assessed according to numerous parameters that are used to determine a neuropsychological conclusion about the state of a subject’s cognitive functions. The system for calculating integral indices has also been developed to determine generalized quantitative indicators of the state of different cognitive processes (Korneev & Akhutina, 2016). Each of those indices consists of a set of performance indicators for different tests within the examination.

At present, our approach applies a set of indices that allows for evaluating the following processes: programming and control of voluntary actions (executive functions); serial organization of movements and speech; processing of kinesthetic information; processing of auditory information; processing of visual information; processing of visual-spatial information; hyperactivity/impulsivity; and fatigue/slow tempo. When diagnosing the state of cognitive functions in preschool and primary school children, that set of indices is quite complete and makes it possible to assess all of the most important cognitive components that intensively develop at that age, and are important for the child’s success in learning (Ardila & Rosselli, 1994; Klenberg, Korkman, & Lahti-Nuuttila, 2001; Korkman, Kemp, & Kirk, 2001; Stiles, Akshoomoff, & Haist, 2013; Vanvooren, Poelmans, De Vos, Ghesquière, & Wouters, 2017). It is important that the indices not indicate global multifactorial constructs like memory or language but rather the component processes that underlie complex cognitive functions in the children.

In recent years, computerized batteries for neuropsychological examinations have been developing rapidly. The most famous of them is CANTAB (Luciana & Nelson, 2002), but there are also several others, including MINDS (Brand, & Houx, 1992; Brand, von Borries, & Bulten, 2010), ANAM, ImPACT, CogState, and CNS-VS (see Parsons, 2016 for a review). We also have developed and used a battery of tests aimed at evaluating executive functions, functions of activation (arousal), and functions for processing visual-spatial and auditory information (Korneev et al., 2018). Our computer battery is based on ideas of the Lurian assessment and provides a more objective and standardized way to estimate cognitive functions in children. It can be used as a method for screening children with a risk of learning or other disabilities. In this battery we apply the same approach when certain cognitive functions are evaluated by integral indices that consist of a set of indicators derived from the performance of several tests. A separate task may be the creation of such integral indicators that combine the performance parameters of both face-to-face and computer-based neuropsychological tests.

The techniques for calculating the indices are described in our previous works (Korneev, & Akhutina, 2016; Akhutina, Korneev, Matveeva, Gusev, & Kremlev, 2019). A detailed description of the procedure is beyond the scope of this paper, but in brief, the main idea is to standardize the parameters (productivities and specific mistakes in various tests) and to sum up the z-values into integral estimations.

Since the elaboration of the indices is based on theoretical analysis and the experience of neuropsychological examinations, suggestions about the composition of the indices should be verified in targeted studies. This current work attempts to verify the structural validity of a set of indices of different cognitive functions that we developed specifically for preschool and primary school children. Confirmatory factor analysis is one of the methods of that assessment. This method is quite common in the study of the structure of cognitive functions. Thus, there are studies of the factor structure of executive functions that allow for identifying and assessing their different components: inhibition, updating, and shifting (Miyake, 2000; Friedman & Miyake, 2017). This approach is also employed in the analysis of the structure of intelligence based on the results of the Wechsler Intelligence Scale for Children, which distinguishes four factors: verbal, perceptual, processing speed, and working memory abilities (Bodin, Pardini, Burns, & Stevens, 2009). Using confirmatory factor analysis, we intend to assess the structural validity of the indices that characterize the state of different cognitive functions in children, while including various indicators of task performance in both paper and pencil and computerized neuropsychological examinations. Our research questions may be formulated in the following way:

1. To what extent can the composition of neuropsychological indices, which were developed earlier and based on the theory and practice of child neuropsychology, be verified on a large sample of typically developing children ages 6 to 9 years old?
2. Is it possible to combine the results of a face-to-face examination and computer tests for a more accurate and reliable assessment of the state of cognitive functions?

## Method

### Participants

This study involved 299 children ages 5.1 to 9.5 years. Of these, 111 were preschoolers (56 boys and 55 girls; average age 6.45 years); 82 were first graders (31 boys, 51 girls; average age 7.67 years); and 106 were second graders (62 boys, 44 girls; average age 8.58 years). The participants were from regular Moscow kindergartens and primary schools; their families had middle socioeconomic status. None of the participants had any diagnosed neurological or developmental disorders. The parents of all children gave informed consent for them to participate in the study.

### Measures

#### Face-to-face neuropsychological examination

The neuropsychological examination adapted for children ages 6 to 9 years old was used to determine the state of their cognitive functions (Akhutina, 2016). Although a comprehensive examination includes 20 behavioral tests, in this study we analyzed data from only some of them. The list of the tests with a short description of the procedure is presented in *Table 1*.

**Table 1:** Test battery for the neuropsychological assessment of children 6-to-9 year-olds (partial)

Test	Description
***Executive functions and Serial organization of movements and speech***	
Go/No Go Task, Reciprocal Motor Program Test	The test consists of two series: 1. One knock should be responded to with two knocks, and two knocks with one knock; 2. One knock should be responded to with two knocks, and two knocks with no knock.
Verbal Fluency Tests	The test consists of three series. The participant must name as many words as he can in one minute: 1. any words; 2. names of actions; 3. names of plants.
Odd one out	Five series of five words are presented aurally. The child has to find the odd one and explain the choice.
Counting	The participant must count from 1 to 10; from 10 to 1; from 3 to 7; from 8 to 4; or count from 20 subtracting 3 at a time.
3 Positions Test, or “Fist-Edge-Palm”	The participant must remember and automate a series of hand-movements: Fist-Edge-Palm
Oseretskii-Luria Test of Reciprocal Coordination (RecC.)	The participant is asked to reverse the configurations of his or her hands repeatedly and simultaneously from palm to fist, so that when the fist is opened in one hand, it is closed in the other.
***Kinesthetic information processing***	
Finger Position Test	The test consists of three series: 1. imitation of finger positions (five positions for each hand); 2. reproduction of finger poses using proprioceptive memory (three positions for each hand); 3. transferring finger poses from one hand to the other (three positions for each hand).
Oral Praxis	The participant must perform movements and poses using the orofacial muscles on verbal command (ten tasks).
***Auditory information processing***	
Verbal Memory Test	The participant must remember two groups of three words each. Three attempts are given, and delayed replay is also evaluated.
***Visual information processing***	
Visuo-perceptual Tests	The participant must identify superimposed, crossed out, and unfinished images (22 pictures).
Design Fluency Tests (DFluency)	The participant must draw any eight objects and any eight plants.
***Visual-spatial information processing***	
Visual-Spatial Memory	Four nonverbal figures are presented for eight seconds. The participant must remember and draw them. Three attempts are given, and delayed replay is also evaluated.
Three-Dimensional Drawings	The child must copy a three-dimensional picture of a house.

Test performance was evaluated by an expert neuropsychologist according to several parameters that indicate the child’s ability to understand the task, the accuracy and efficiency of performance, and specific mistakes revealing any weakness of the tested function (primary mistakes) and other functions (secondary mistakes). We calculated productivity as the number of correct answers, marks of accuracy in ordinal scales (from 0 to 3), and numbers of specific or unspecific mistakes. For instance, when analyzing the Finger Position test, kinesthetic difficulties (specifically the prolonged search for a pose) were assessed as primary errors and impulsive performance as a secondary error.

According to the results of the examination, the neuropsychologist could evaluate the state of the functions of activation: symptoms of fatigue, sluggishness, a tendency to perseveration, impulsivity, and hyperactivity (on an ordinal scale from 0 to 3).

#### Computerized neuropsychological tests

In this study, we used tests from the battery of the Computerized Neuropsychological Assessment in 6-9 Year-old Children (Korneev, Akhutina, Gusev, Kremlev, & Matveeva, 2018). The battery consists of 10 tests; five of them were used in the present work:

1. The Dots test (Davidson, Amso, Anderson, & Diamond, 2006). The test consists of three subtests; each of them involves 20 stimuli. In the first subtest, the stimuli (hearts) are presented on the computer screen, in a quasi-random order, to the left or to the right of the screen center. The child’s task is to press the button on the side where the stimulus appears as quickly as possible. The subtest assesses the ability of the participant to follow instructions and the speed of a simple motor reaction. The second subtest evaluates the child’s ability to inhibit the “natural” response that is irrelevant to the task: another stimulus (a flower) appears on the screen; the task is to press the button as quickly as possible on the side opposite to the one where the stimulus appears. The third subtest evaluates the child’s ability to switch between the two parallel programs: two types of stimuli (hearts and flowers) are presented alternately on the screen; the task is to press the key on the same side where the heart appears, and on the opposite side where the flower appears. This test is assessed according to the average response time and productivity (the number of correct responses).
2. A computer version of the “Schulte Tables” (Korneev et al., 2018). The test consists of five subtests; each presents a table consisting of 20 cells on a touch screen. In those cells, there are two series of numbers from 1 to 10 arranged in quasi-random order; one series consists of black numbers, while the second set of numbers is red. The participant must search for and indicate the numbers in a certain order by touching the screen with a finger. The first subtest calls for pointing to the black numbers from 1 to 10, followed by the red numbers from 1 to 10 in the second subtest; then in the third subtest, the black numbers from 10 to 1; in the fourth subtest, there should be two parallel series showing red and black numbers in ascending order (1 black, 1 red, 2 black, 2 red, etc.), and in the fifth, the participant must indicate red numbers from 10 to 1. Such a set of tasks makes it possible to assess the children’s ability to master a simple action program (the first and second subtests), a more complicated reverse program (the third and fifth subtests), and the most difficult, a “parallel” action program (the fourth subtest). They have to switch their attention from one program to another and must inhibit inadequate responses. Based on the results, we calculated the average time of searching for a number as well as the error count, both for the whole test and in the five subtests separately.
3. The Cancellation test for preschoolers and primary school children (Korneev et al., 2018). The test consists of three subtests. The touch screen displays a table consisting of six similar elements (geometric figures in the version for preschoolers, letters in the version for young students). In the first subtest, the child’s task is to find and mark one of the stimuli, in the second one they find and mark the other one, and in the third subtest the target is both of those stimuli. Thus, during the first two trials, we assess the child’s ability to keep their attention on a simple task for quite a long time, and in the third trial, the ability to switch to a more complicated instruction is assessed. The evaluated parameters are tempo (the number of correct answers per minute) and accuracy (the percentage of correct answers).
4. The Corsi Tapping Block test (Milner, 1971; a computerized version for children by Korneev et al., 2018). Nine cubes presented on the touch screen light up one by one. The task is to remember their positions on the screen, and after their presentation, the participant must reproduce the sequence of the highlighted cubes. The trial starts with a row of two cubes; with every right answer, the length of the row increases. The indicators in this trial are the maximum length of a correctly reproduced sequence, the average time of the first response, and the average time of pauses within the sequence.
5. The Understanding of Similar Sounding Words test (USSW; Korneev et al., 2018). The child is presented with a set of 10 pictures of distinct objects whose names differ in one sound; for instance, “bochka-pochka” (barrel-bud). Then a sequence of words is presented aurally (a total of eight sequences, each two to Thve words in length). The child must indicate the corresponding pictures in the same order. We evaluate the percentage of correctly reproduced words (relative to the total number of responses) and the numbers of different mistakes (substitutions of similar and dissimilar sounding words and omissions).

### Analysis

Confirmatory factor analysis was used to test our hypothesis about the possibility of identifying the parameters that characterize different groups of cognitive functions. The parameters of the tests’ performance (productivity, specific errors, and reaction time) were used as the exogenous variables (indicators) in the model, and the cognitive functions were included as endogenous variables (factors). Since some of the performance indices of the neuropsychological examination were estimated on ordinal scales, we used the method of weighted least squares with the means and variation adjusted (WLSMV; Muthén & Muthén, 2012). This method is applied in the case of ordinal scales and is resistant to a non-normal distribution of data. The analysis was conducted in R version 3.6.0 with the Lavaan package (ver. 0.6-9, Rosseel, 2012). To assess the quality of the models, we used the following rules: for CFIand TLI, values higher than .90 reflect a good model fit; for RMSEA, less than .08 indicates close fit (Schumacker, & Lomax, 2010).

## Results

### Models with parameters of face-to-face assessment

Several models were constructed and tested. The first model corresponded to the composition of indices used in practice (Korneev & Akhutina, 2016). It included eight factors:

1. Executive functions (hereafter EF): the total number of mistakes, performance speed, and understanding the instruction in the second part of the Go/ No go test; performance in the first two parts of the verbal fluency tests (VFT) and number of inadequate responses in the third part of the VFT; number of errors in the Counting test; mastering the program in the 3 Positions test; and productivity, number of inadequate responses, and overall score on the Odd One Out test;
2. Serial organization of movements (SerOrg): mastering the program, quality of execution, and number of errors in the 3 Positions test; performance on the Reciprocal Coordination test;
3. Kinesthetic information processing (Kinest): performance on the Finger Positions test, efficiency of reproduction by the left hand according to the proprioceptive pattern, efficiency of the pattern transfer, number of kinesthetic errors, and productivity in the Oral Praxis test;
4. Auditory information processing (Aud): productivity of repetition and reproduction after the third presentation in the Verbal Memory test, substitutions of one consonant or a vowel sound when reproducing words, efficiency of the second part of the Verbal Fluency test, number of verbal mistakes in the Visual Perception test;
5. Visual information processing (Vis): productivity, number of visual errors in the Visual Perception test, number of well-recognized pictures in the Design Fluency tests, and the tree drawing score in 3-Dimensional Drawing;
6. Visual-spatial information processing (VSp): productivity of the first and third reproduction of stimuli in the Visual-Spatial Memory test, number of transformations of stimuli into a sign, severity of weakness of the right-hemisphere or left-hemisphere strategy in 3-Dimensional Drawing, and number of spatial mistakes in the Finger Positions test;
7. Sluggish tempo (ST): indices of fatigue, a lower tempo of task performance, and severity of the tendency to perseveration; and
8. Hyperactivity/Impulsivity (HImp): indices of hyperactivity and impulsivity.

The model also allowed for correlations between all those factors. The estimates of this model turned out to be acceptable but not too high (x2(915) = 2295.921, CFI= 0.907, TLI = 0.899, RMSEA = 0.071); the full data on the coefficients of the model are given in the Appendix, *Table 1A*. However, since some variables in this model had very small factor loads, we modified the model to exclude such cases. On the EF factor index, the index of mastering in the 3 Positions test was excluded. On the Kinest factor, performance in the Oral Praxis test was excluded (this index displays the ceiling effect). On the Aud factor, we excluded the vowel change in the Verbal Memory test (this is a rare error) and verbal errors in the Visual Perception test. On the Visual-spatial factor, the number of transformations into a sign in the Visual-spatial Memory test and spatial errors in the Finger Positions test were excluded.

Meanwhile, the productivity of the first repetition and the number of distortions during the reproduction of words in the Verbal Memory test were added to the factor of processing the auditory information. The estimates of this modification improved ((X2(750) = 1692.926, CFI= 0.918, TLI = 0.910, RMSEA = 0.065; the full data about the coefficients of the model are given in the Appendix, *Table 2A*), and all of the factor loads were significantly different from zero at the level of p < 0.05.

### Model with parameters of both face-to-face and computerized assessment

The addition of the indices of computer test performance into the model was the next step. The following were added:

1. to factor EF: productivity of the third subtest of Dots, number of mistakes in the fourth Schulte Table, and the total accuracy of the Cancellation test;
2. to factor Aud: productivity and similar replacements in the USSW;
3. to factor VSp: productivity in the Corsi Test and response time (search) in the fourth Schulte Table;
4. to the factors of sluggishness and hyperactivity: response times in the first subtests of Dots and Schulte Table, the average interval between responses in the Corsi Test, and the average tempo in the Cancellation test.

The estimates of that model improved (X2(1241) = 2579.507, CFI= 0.935, TLI = 0.930, RMSEA = 0.060); the full data on the model coefficients are given in *Table 2*.

**Table 2:** Structural model coefficients

Factor	Test	Variable	Est.	error Std.	Z–value	Sig.
**EF**	Go/ No go	understanding forthe second the trial instructions	0.550	0.062	8.825	<0.001
		number of errors	0.669	0.025	27.241	<0.001
		speed	0.339	0.039	8.592	<0.001
	Verb. Fluency	VF 1 — productivity	–0.555	0.040	–14.003	<0.001
		VF 2 — productivity	–0.313	0.068	–4.577	<0.001
		VF 3 — inadequate responses	0.177	0.057	3.121	0.002
	Counting	availability	0.466	0.030	15.544	<0.001
		number of errors	0.302	0.053	5.740	<0.001
	Odd One Out	productivity	–0.584	0.037	–15.918	<0.001
		total score	–0.537	0.044	–12.086	<0.001
		inadequate responses	0.431	0.035	12.172	<0.001
	Dots	productivity in Dots 3	–0.480	0.045	–10.723	<0.001
	Schulte	errors in Schulte 4	0.195	0.053	3.656	<0.001
	Cancel.	accuracy in the Cancellation test	–0.296	0.052	–5.702	<0.001
**SerOrg**	3 Positions test	mastering program	0.586	0.047	12.356	<0.001
		productivity	0.849	0.047	18.087	<0.001
		errors in serial organization	0.572	0.053	10.900	<0.001
	Rec. c.	productivity	0.638	0.048	13.157	<0.001
**Kinest**	Finger position test	productivity acc. to the proprioceptive pattern	–0.700	0.061	–11.519	<0.001
		productivity in the transfer	–0.704	0.049	–14.418	<0.001
		kinesthetic errors in total	0.956	0.046	20.564	<0.001
		performance rate	0.483	0.065	7.396	<0.001
**Aud**	VFluen. Verbal Memory	VF 2 — productivity	–0.339	0.080	–4.256	<0.001
		productivity in repetition 1	–0.534	0.049	–10.851	<0.001
		productivity in repetition 3	–0.591	0.052	–11.381	<0.001
		productivity in reproduction 3	–0.685	0.047	–14.593	<0.001
		substitution of the first consonant	0.240	0.065	3.676	<0.001
		distortions	0.282	0.060	4.673	<0.001
	Underst.	productivity	–0.596	0.064	–9.263	<0.001
	sim.s.w.	similar errors	0.354	0.133	2.662	0.008
**Vis**	Visual perception	productivity superimposed of pictures the recognition of	–0.596	0.041	–14.563	<0.001
		productivity unfinished pictures of the recognition of	–0.688	0.036	–19.008	<0.001
		perceptually similar mistakes	0.311	0.054	5.807	<0.001
		rough errors	0.535	0.045	11.952	<0.001
		fragmentary errors	0.278	0.060	4.634	<0.001
		errors in shape/background	0.310	0.049	6.286	<0.001
	DFluency	well-recognized pictures	–0.556	0.052	–10.685	<0.001
	3 dim. dr.	picture of a tree	0.766	0.028	27.544	<0.001
**VSp**	Visual- spatial memory	productivity in reproduction 1	–0.442	0.043	–10.340	<0.001
		productivity in reproduction 3	–0.523	0.043	–12.266	<0.001
	3 dim. drawing	holistic strategy	0.687	0.035	19.428	<0.001
		analytic strategy	0.674	0.023	29.419	<0.001
	Corsi	productivity	–0.443	0.047	–9.509	<0.001
	Schulte	errors in Schulte 4	0.705	0.021	33.772	<0.001
**ST**	Obser-vations	fatigue	0.623	0.055	11.427	<0.001
		slow tempo	0.281	0.048	5.818	<0.001
		tendency to perseveration	0.457	0.057	8.032	<0.001
	Schulte	time in Schulte 1	0.851	0.088	9.642	<0.001
	Dots	time in Dots 1	0.495	0.092	5.389	<0.001
	Corsi	time in Corsi	0.330	0.069	4.765	<0.001
	Cancel.	tempo in the Cancellation test	–0.292	0.073	–3.994	<0.001
**HImp**	Obser-vations	impulsivity	0.720	0.063	11.341	<0.001
		hyperactivity	0.992	0.085	11.652	<0.001
	Schulte	time in Schulte 1	–0.720	0.111	–6.470	<0.001
	Dots	time in Dots 1	–0.468	0.102	–4.594	<0.001
	Corsi	time in Corsi	–0.446	0.080	–5.556	<0.001
	Cancel.	tempo in the Cancellation test	0.280	0.078	3.608	<0.001

*Note. Est. = estimated coefficients, std. err. = standard errors, sig. = significance of t-test*.

The correlations between the factors are shown in *Table 3*.

**Table 3:** Correlations between the factors in the model

	SerOrg	Kinest	Aud	Vis	VSp	ST	HImp
EF	0.725*	0.302*	0.566*	0.787*	0.919*	0.793*	0.119
SerOrg		0.288*	0.341*	0.579*	0.658*	0.510*	0.192*
Kinest			0.161*	0.312*	0.395*	0.307*	0.128
Aud				0.505*	0.570*	0.251*	–0.162*
Vis					0.886*	0.637*	0.136
VSp						0.718*	0.014
ST							0.613*

Note. * = significant at level p < 0.05

## Discussion

A confirmatory factor analysis of the performance parameters of the various tests from the batteries of face-to-face and computer neuropsychological examination made it possible to identify the factor structure that corresponded to the proposed structure of integral estimates of different groups of cognitive functions.

We detected and confirmed two factors associated with the functions of activation at the level of empirical data. These were the hyperactivity/impulsivity factor, which correlated with disturbances in the form of ADHD (Barkley, 1998), and the factor of sluggishness, which manifested in the syndrome of a sluggish cognitive tempo (Becker, Marshall, & McBurnett, 2014; Becker & Willcutt, 2019). These results correspond to the data obtained in fMRI research (Fassbender, Krafft , & Schweitzer, 2015). It is worth emphasizing that, in our model, the same indices of performance times for the computer tests had significant loads in both factors but with an opposite sign. This also justifies separating the neurodynamic functions in that way.

As to the functions associated with information processing, we received confirmation at the level of the structural model for the validity of the division of individual factors for the processing of kinesthetic, auditory, visual, and visual-spatial information. The possibility of identifying modally specific mechanisms of information processing in solving different tasks is under discussion in the literature. There are arguments both in favor of (Barsalou, Simmons, Barbey, & Wilson, 2003) and against (Anderson, Qin, Jung, & Carter, 2007) that division. Within the framework of our approach, the results obtained on a sample of typically developing children pointed toward such a division, at least at the level of behavioral indicators of neuropsychological task performance.

As to the distinction of factors connected with voluntary activity, following A.R.Luria (1976, 1980) in this case, our model distinguished the factor of the programming and control of activity (~ executive functions) and the factor of the serial organization of actions. The results of the confirmatory analysis also reaffirmed that division. Executive functions are important for the performance of almost any voluntary activity; some studies show that they have a heterogeneous structure and can be divided into separate groups of functions (inhibiting, updating, and shifting; see Miyake et al., 2000; Friedman & Miyake, 2017). Such detailing was not carried out in our research; it may require a separate study based on neuropsychological examination.

It is noteworthy that the identified factors highly correlate with each other. This is not surprising, as it is difficult to expect them to be independent of each other. Solving even a simple task inevitably engages several groups of functions. This corresponds to ideas about the relationships between functions in neuropsychological theory. We attempted to identify the indices of the states of certain specific functions, and we managed to do that to some extent, although at the same time we detected quite a close relationship between functions. Whether it is possible to identify more “pure” indicators of certain functions within the framework of ecologically valid tasks in the examination is a topic for further discussion. Such a “targeted” assessment is possible through more specific laboratory studies held in the framework of experimental psychology.

Another significant result obtained in our study was the preservation of, and even some improvement in, the quality of the model by including the results of the computer tests. This indicates that the combined usage of face-to-face and computerized neuropsychological examinations can improve the reliability and accuracy of the assessment of the states of different functions. There are data about a weak correlation between the results from the computer tests and the traditional neuropsychological examination (Smith, Need, Cirulli, Chiba-Falek, & Attix, 2013). Our model detected that the same indices (reaction time) may be associated with different factors with an opposite sign. This is explainable and meaningful, but it can also be the reason for the weakening of simple linear relationships between the results of different methods. Constructing and testing models like the one described in our paper can be an efficient way to investigate the consistency of the tests.

Thus, the results of our confirmatory analysis support the structural validity of identifying our hypothesized groups of functions and the validity of evaluating them by indices that include indicators of the performance of different tasks within the neuropsychological examination. This approach makes it possible to carry out a detailed and comprehensive analysis of the cognitive states of children at preschool and primary-school ages, with typical development and with different disorders. In the typical samples, this approach makes it possible to distinguish children with a relative weakness (deficiency) of certain abilities associated with the uneven development of some functions and, if necessary, to arrange a preventive remedial intervention. With more pronounced behavioral problems classified as disorders, such an assessment provides an opportunity to analyze the structure of the defect and to suggest the most efficient ways to correct it.

## Conclusions

The present study proposed and evaluated several models describing the possible composition of indices of different cognitive functions, made up of indicators of performance on neuropsychological examinations by preschool and primary school children. They include the following groups of cognitive functions: programming and control of voluntary actions (executive functions), serial organization of movements and speech; the processing of kinesthetic information; the processing of auditory information; the processing of visual information; the processing of visual-spatial information; hyperactivity/impulsivity; and fatigue/slow tempo. The final model showed good consistency with the data. This confirmed the structural validity of the proposed scheme for quantifying the state of the cognitive sphere in children.

Including the performance indices from two methods (face-to-face and computerized tests) had the important result of improving the quality of the model. Furthermore, this model may be useful in the research of the patterns of cognitive capacities in children with typical and deviant developments. Secondly, our findings make it possible to construct more detailed models that clarify the structure of cognitive functions, especially executive functions, in children. In general, our work shows that the qualitative approach to neuropsychological diagnostics, developed by A.R. Luria (Luria, 1980), can be effectively combined with quantitative analysis of neuropsychological data.

## Limitations

Our study had some limitations. We did not analyze the influence of socioeconomic factors, although they may be important. We selected schools with approximately the same average socioeconomic level of children, but more detailed and precise analysis may be needed in future studies. Our sample included only children of the metropolis, so it would be important to test our findings on samples from other regions.

Co mputer methods allow us to see the quantitative characteristics of some cognitive functions, but they still do not replace expert qualitative assessments. Computer tests can be used as a screening method: to identify children with risk for learning disabilities. The quantitative estimates obtained with their help can complement the qualitative assessment of the expert.

We have developed and discussed the integrative indices that can be useful in the situation of screening or for the generalization of results of the assessment in large samples. But this approach is less sensitive than a neuropsychological conclusion made by an expert. We have to remember that both the qualitative and quantitative approaches have both strengths and weaknesses.

## Appendix

**Table 1A:** The structural model coefficients for Model 1

Factor	Test	Variable	Est.	error Std.	Z–value	Sig.
**EF**	Go - no go	understanding the instruction for the second trial	0.529	0.061	8.681	<0.001
		number of errors	0.530	0.019	27.292	<0.001
		speed	0.410	0.046	8.830	<0.001
	Verb. Fluency	VF 1— productivity	–0.502	0.048	–10.561	<0.001
		VF 2— productivity	–0.121	0.073	–1.658	0.097
		VF 3— inadequate responses	0.169	0.058	2.910	0.004
	Counting	availability	0.514	0.041	12.586	<0.001
		number of errors	0.358	0.056	6.429	<0.001
	Odd one out	productivity	–0.668	0.030	–22.351	<0.001
		total score	–0.930	0.025	–37.787	<0.001
		inadequate responses	0.801	0.021	37.677	<0.001
	3 positions test	Mastering program	0.056	0.069	0.814	0.416
**SerOrd**	3 positions test	Mastering program	0.563	0.090	6.262	<0.001
		productivity	0.807	0.047	17.018	<0.001
		errors in the serial organization	0.589	0.052	11.338	<0.001
	Rec. c.	productivity	0.658	0.050	13.199	<0.001
**Kinest**	Finger position test	productivity acc. to the proprioceptive pattern	–0.634	0.061	–10.432	<0.001
		productivity in the transfer	–0.720	0.048	–14.925	<0.001
		kinesthetic errors in total	0.942	0.047	20.111	<0.001
		performance rate	0.555	0.065	8.514	<0.001
	Oral Praxis	Productivity	–0.015	0.081	–0.187	0.851
**Aud**	VFluen	VF 2— productivity	–0.556	0.083	–6.738	<0.001
	Visual perception	Verbal errors in the recognition of the superimposed pictures	0.030	0.077	0.398	0.691
		Verbal errors in the recognition of the unfinished pictures	0.095	0.071	1.338	0.181
		productivity in repetition 3	–0.616	0.049	–12.666	<0.001
		productivity in reproduction 3	–0.583	0.056	–10.404	<0.001
		substitution of the 1st consonant	0.180	0.069	2.600	0.009
		substitution of the 1st vowel	0.083	0.081	1.019	0.308
**Vis**	Visual percep- tion	productivity of the recognition of the superimposed pictures	–0.585	0.045	–13.123	<0.001
		productivity the unfinished of the pictures recognition of	–0.654	0.041	–15.821	<0.001
		perceptually similar mistakes	0.273	0.059	4.635	<0.001
		rough errors	0.508	0.046	11.120	<0.001
		fragmentary errors	0.292	0.059	4.976	<0.001
		errors in the shape/background	0.320	0.054	5.878	<0.001
	DFluency	well-recognized pictures	–0.546	0.053	–10.360	<0.001
	3 dim. dr.	picture of a tree	0.822	0.032	25.556	<0.001
**VSp**	Visual-spatial memory	productivity in reproduction 1	–0.452	0.051	–8.833	<0.001
		productivity in reproduction 3	–0.537	0.046	–11.692	<0.001
		Transformation to a sign	0.039	0.051	0.772	0.440
	3 dim. drawing	holistic strategy	0.714	0.035	20.459	<0.001
		analytic strategy	0.789	0.037	21.099	<0.001
	Finger test position	Spatial errors	0.074	0.058	1.280	0.201
**ST**	Observations	fatigability	0.891	0.076	11.740	<0.001
		slow tempo	0.434	0.065	6.702	<0.001
		tendency to perseveration	0.597	0.071	8.449	<0.001
**HImp**	Observations	impulsivity	0.759	0.101	7.500	<0.001
		hyperactivity	0.981	0.129	7.607	<0.001

*Note. Est. = estimated coefficients; std. err. = standard errors; sig. = significance of t-test*.

**Table 2A:** The structural model coefficients for Model 2

Factor	Test	Variable	Est.	error Std.	Z–value	Sig.
**EF**	Go - no go	understanding the instruction for the second trial	0.553	0.065	8.459	<0.001
		number of errors	0.680	0.032	21.123	<0.001
		speed	0.361	0.040	8.961	<0.001
	Verb. Fluency	VF 1— productivity	–0.522	0.049	–10.736	<0.001
		VF 2— productivity	–0.315	0.076	–4.134	0.097
		VF 3— inadequate responses	0.188	0.057	3.297	0.004
	Counting	availability	0.472	0.037	12.925	<0.001
		number of errors	0.338	0.054	6.262	<0.001
	Odd one out	productivity	–0.592	0.039	–15.175	<0.001
		total score	–0.518	0.048	–10.772	<0.001
		inadequate responses	0.448	0.047	9.456	<0.001
**SerOrd**	3 positions test	Mastering program	0.595	0.048	12.397	<0.001
		productivity	0.824	0.047	17.487	<0.001
		ewrrors in the serial organization	0.588	0.051	11.500	<0.001
	Rec. c.	productivity	0.646	0.047	13.834	<0.001
**Kinest**	Finger position test	productivity acc. to the proprioceptive pattern	–0.641	0.061	–10.450	<0.001
		productivity in the transfer	–0.721	0.049	–14.754	<0.001
		kinesthetic errors in total	0.939	0.047	19.847	<0.001
		performance rate	0.552	0.067	8.277	<0.001
**Aud**	VFluen.	VF 2— productivity	–0.298	0.081	–3.660	<0.001
	Visual percep- tion	productivity in repetition 1	–0.626	0.048	–13.046	0.691
		productivity in repetition 3	–0.685	0.048	–14.214	<0.001
		productivity in reproduction 3	–0.708	0.047	–14.951	<0.001
		substitution of the 1st consonant	0.274	0.060	4.598	0.009
		Distortions	0.306	0.054	5.616	0.308
**Vis**	Visual percep- tion	productivity of the recognition of the superimposed pictures	–0.581	0.045	–12.797	<0.001
		productivity of the recognition of the unfinished pictures	–0.652	0.042	–15.604	<0.001
		perceptually similar mistakes	0.281	0.060	4.669	<0.001
		rough errors	0.506	0.046	11.081	<0.001
		fragmentary errors	0.291	0.059	4.962	<0.001
		errors in the shape/background	0.333	0.054	6.176	<0.001
	DFluency	well-recognized pictures	–0.550	0.053	–10.400	<0.001
	3 dim. dr.	picture of a tree	0.818	0.032	25.592	<0.001
**VSp**	Visual-spatial memory	productivity in reproduction 1	–0.457	0.051	–9.017	<0.001
		productivity in reproduction 3	–0.551	0.046	–12.031	<0.001
	3 dim. drawing	holistic strategy	0.720	0.035	20.326	<0.001
		analytic strategy	0.770	0.037	20.897	<0.001
**ST**	Observations	fatigability	0.905	0.076	11.843	<0.001
		slow tempo	0.400	0.061	6.585	<0.001
		tendency to perseveration	0.602	0.071	8.418	<0.001
**HImp**	Observations	impulsivity	0.698	0.083	8.376	<0.001
		hyperactivity	1.067	0.127	8.397	<0.001

*Note. Est. = estimated coefficients; std. err. = standard errors; sig. = significance of t-test*.
